# Association of cerebral oxygenation with estimated glomerular filtration rate and cognitive function in chronic kidney disease patients without dialysis therapy

**DOI:** 10.1371/journal.pone.0199366

**Published:** 2018-06-25

**Authors:** Haruhisa Miyazawa, Susumu Ookawara, Kiyonori Ito, Yuichiro Ueda, Katsunori Yanai, Hiroki Ishii, Yuko Mutsuyoshi, Taisuke Kitano, Mitsutoshi Shindo, Akinori Aomatsu, Keiji Hirai, Taro Hoshino, Yoshiyuki Morishita

**Affiliations:** Division of Nephrology, First Department of Integrated Medicine, Saitama Medical Center, Jichi Medical University, Saitama, Japan; Osake University Graduate School Of Medicine, JAPAN

## Abstract

**Background:**

A decline in estimated glomerular filtration rate (eGFR) is reportedly associated with increased prevalence rates of cognitive impairment. However, data concerning the association between the cerebral saturation of oxygen (rSO_2_) and cognitive function of patients with chronic kidney disease (CKD) is limited. This study aimed to (i) elucidate the clinical factors associating with cerebral rSO_2_ and (ii) investigate the association between cerebral rSO2 and cognitive assessment in CKD patients.

**Methods:**

A total of 40 CKD patients not requiring dialysis (26 men and 14 women; mean age, 61.0 ± 2.7 years) were recruited. The numbers of patients at each CKD stage were as follows: G1, 5; G2, 8; G3a, 6; G3b, 5; G4, 11; and G5, 5. Cerebral rSO_2_ was monitored at the forehead using the oxygen saturation monitor INVOS 5100C. The cognitive function of each patient was confirmed using the Mini-Mental State Examination (MMSE).

**Results:**

Cerebral rSO_2_ levels were significantly higher in CKD patients than in hemodialysis patients (63.8 ± 1.5% vs. 44.9 ± 2.2%, *p* < 0.001). Multiple regression analysis showed that cerebral rSO_2_ was independently associated with eGFR (standardized coefficient: 0.530), serum albumin concentration (standardized coefficient: 0.365), and serum sodium concentration (standardized coefficient: 0.224). Furthermore, MMSE showed a significantly positive correlation with cerebral rSO_2_ (r = 0.624, *p* < 0.001).

**Conclusions:**

Cerebral rSO_2_ was affected by eGFR and serum albumin and sodium concentrations in CKD patients. Furthermore, cerebral rSO_2_ monitoring, which reflected MMSE scores, might be a useful method for assessing cognitive function in CKD patients.

## Introduction

Cognitive impairment and dementia have been recognized as complications in patients with chronic kidney disease (CKD), including those undergoing hemodialysis (HD), and a decline in estimated glomerular filtration rate (eGFR) was associated with increased prevalence rates of cognitive impairment among adults in the United States [1]. Therefore, it is preferable to start the screening of cognitive function before the onset of end stage renal disease [2]. Studies have reported many screening methods for the detection of cognitive function; however, several tests involve difficulties in accurately detecting the cognitive function because of their low sensitivities and complexities to perform [3–6]. In addition, no instruments are available for evaluating the cognitive function of CKD patients, including those undergoing dialysis therapy [2]. Therefore, new simple methods using instruments are required in clinical settings. Recently, near-infrared spectroscopy (NIRS) has been used as a tool to measure the regional saturation of oxygen (rSO_2_), a marker of tissue oxygenation, at the frontal cerebral cortex in various clinical situations and has shown a change in critical balance between arterial oxygen delivery and cerebral oxygen consumption [7–10]. Cerebral rSO_2_ was reported to be significantly lower in HD patients than in healthy controls [11–13]. Moreover, these values were associated with pH, HD duration, serum albumin concentration, and the presence of diabetes mellitus (DM) [13]. However, to date, few reports have investigated the relationship between cerebral oxygenation using NIRS and clinical parameters in CKD patients without dialysis therapy, and data is limited on the association between cerebral rSO_2_ and cognitive function of those patients. Therefore, this study aimed to (i) elucidate the clinical factors associated with cerebral rSO_2_ and (ii) investigate the association between cerebral rSO_2_ and cognitive assessment of CKD patients without dialysis therapy.

## Methods

In this single-center observational study, CKD patients who met the following criteria were enrolled: (1) all-stage CKD patients not yet requiring dialysis therapy and were followed-up at the division of nephrology of our hospital and (2) patients who were older than 20 years. The exclusion criteria were the following comorbidities: congestive heart failure, chronic obstructive pulmonary disease, apparent neurological disorder, and chronic hypotension (defined as systolic blood pressure < 100 mmHg).

A total of 40 CKD patients were recruited (26 men and 14 women; mean age, 61.0 ± 2.7 years). The numbers of patients at each CKD stage were as follows: G1, 5; G2, 8; G3a, 6; G3b, 5; G4, 11; and G5, 5. The causes of CKD were chronic glomerulonephritis (n = 24), nephrosclerosis (n = 8), DM (n = 2), and others (n = 6). The patients’ general characteristics are summarized in Table 1. All patients signed informed consent to participate in this study. This study was approved by the Institutional Review Board of Saitama Medical Center, Jichi Medical University, Japan (DAI-RIN 15–104) and conforms to the provisions of the Declaration of Helsinki (as revised in Tokyo in 2004). In addition, as described in detail previously [13,14], 33 patients undergoing HD with more than 6 months after HD initiation (26 men and 7 women; mean age, 65.6 ± 2.0 years; HD vintage, 8.2 ± 1.3 years; cause of end stage renal failure, chronic glomerulonephritis [n = 9]; DM [n = 14]; nephrosclerosis [n = 4]; others [n = 6]) were recruited as the HD group.

### Evaluation of patient’s renal function

The renal function of each patient was evaluated using eGFR based on the serum creatinine concentration (S-Cr). eGFR was calculated using the following equation [15]:

$$\begin{array}{l} eGFR\left(mL/min/1.73m^{2}\right)\;194\times S-Cr^{-1.094}\times age^{-0.287}\left(for\;men\right)\\ =194\times S-Cr^{-1.094}\times age^{-0.287}\times 0.739\;\left(for\;women\right) \end{array}$$

### Monitoring of cerebral oxygenation and clinical laboratory measurement

Cerebral rSO_2_ was monitored at the forehead using an INVOS 5100C saturation monitor (Covidien Japan, Tokyo, Japan), which utilizes NIRS technology. This instrument uses a light-emitting diode, which transmits near-infrared light at 2 wavelengths (735 and 810 nm), and 2 silicon photodiodes, which act as light detectors to measure oxygenated hemoglobin (Hb) and deoxygenated Hb. The ratio of O_2_ Hb to total Hb (oxygenated Hb + deoxygenated Hb) signal strength, which is the corresponding percentage, is read as a single numerical value that represents the rSO_2_ [16,17]. All data obtained by this instrument were immediately and automatically stored in sequence. The interobserver variance for this instrument, namely the reproducibility of the rSO_2_ measurement, is acceptable as previously reported [18–20]. Therefore, rSO_2_ is considered reliable when estimating the actual cerebral oxygenation. Furthermore, the light paths leading from the emitter to the different detectors share a common part; 30-mm detector assesses superficial tissues, while the 40-mm detector is used to assess deep tissues. By analyzing the differential signals recorded by the different detectors, the current data for cerebral rSO_2_ was supposed to be obtained in deep tissue 20–30 mm from the body’s surface [21,22]. These measurements were performed at every 6-second interval. Before the measurement, the recruited patients rested in the supine position for at least 10 min to reduce the influence of postural change [23]. An rSO_2_ measurement sensor was attached to the patient’s forehead for measurement in the resting state. Thereafter, rSO_2_ was measured for 5 min, and the mean cerebral rSO_2_ was evaluated for 5 min, as a marker of cerebral oxygenation, in each patient. Blood and urinary samples were obtained from each patient in addition to the measurement of oxygen saturation (SpO_2_: %) under room air.

### Method of cognitive assessment

Cognitive assessment was confirmed using the Mini-Mental State Examination (MMSE) [6] in each enrolled CKD patient because this test has been popular to evaluate global cognitive function in the clinical setting [24].

## Statistical analysis

Data are expressed as mean ± standard error (SE) or median (interquartile range), as appropriate. Two variables (C-reactive protein and urinary protein excretion) did not have a normal distribution, and these variables were transformed using the natural log (Ln). Correlations between the 2 groups were evaluated by Pearson’s correlation coefficient and linear regression analysis. Student’s t-test for non-paired values was used for comparison between cerebral rSO_2_ values in the CKD groups and those in the HD groups. Variables that were significantly correlated with cerebral rSO_2_ in a simple linear regression analysis were included in the multivariable linear regression analysis to identify factors affecting cerebral rSO_2_ in CKD patients. *p* < 0.05 was considered statistically significant.

## Results

**Fig 1:**
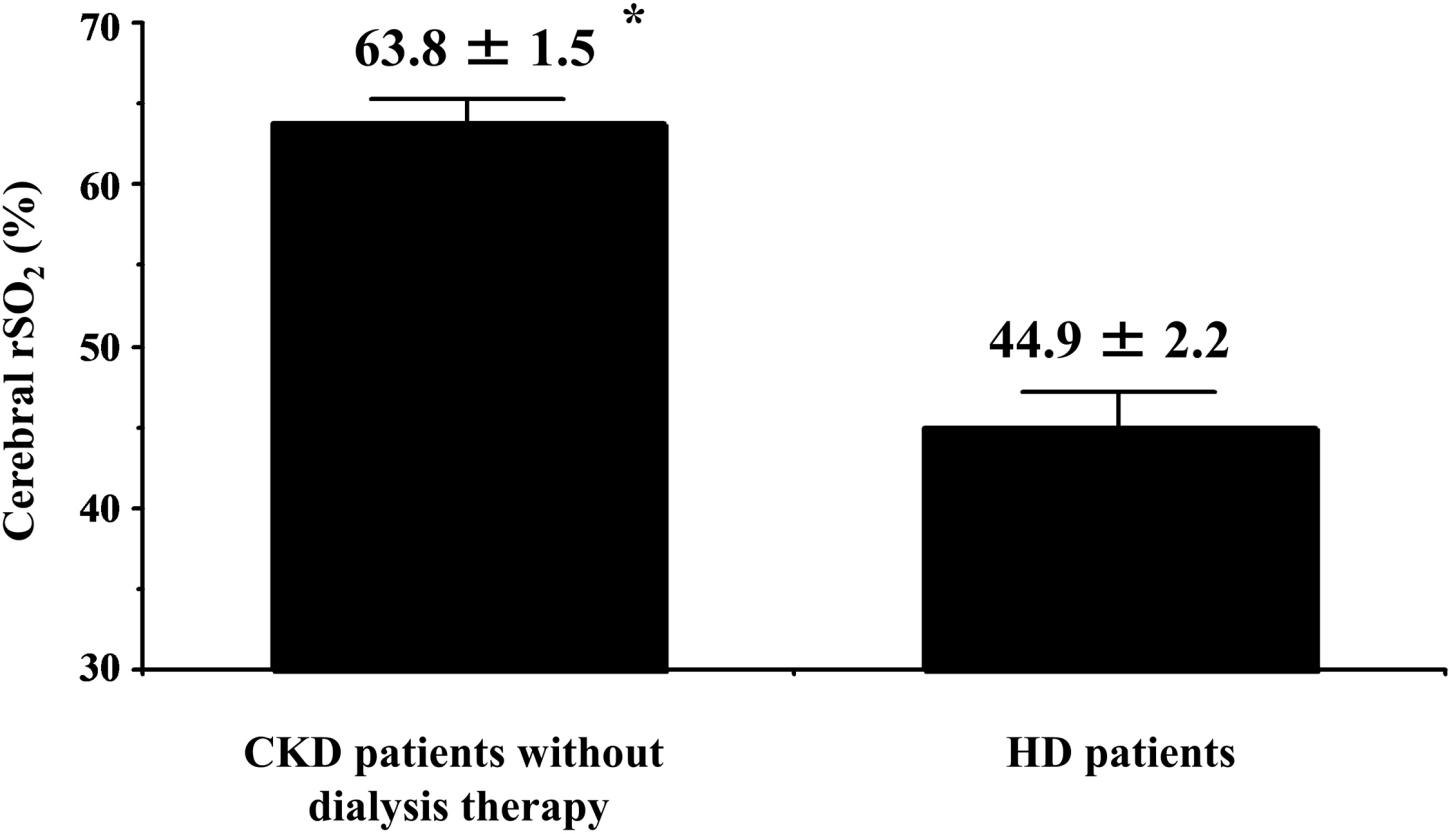
Comparison between cerebral rSO_2_ in CKD patients without dialysis therapy and hemodialysis patients. rSO2, regional saturation of oxygen; CKD, chronic kidney disease; HD, hemodialysis. * p < 0.001 vs. HD patients.

Cerebral rSO_2_ at rest in CKD patients was compared with that in HD patients, and a significant difference was observed between the 2 groups (CKD patients, 63.8 ± 1.5%; HD patients, 44.9 ± 2.2%; *p* < 0.001; Fig 1).

**Fig 2:**
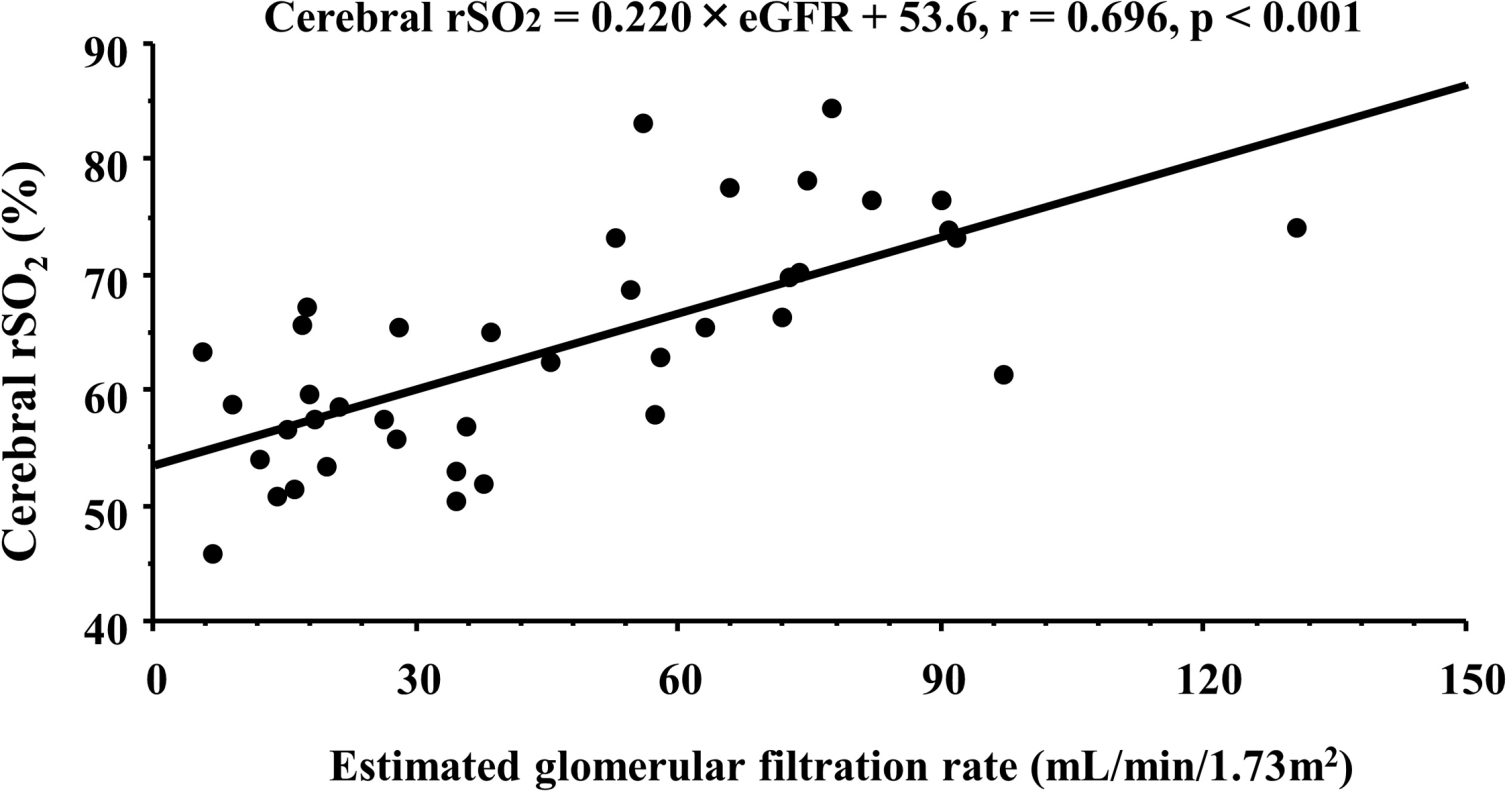
Correlation between cerebral rSO_2_ and eGFR in CKD patients. rSO_2_, regional saturation of oxygen; CKD, chronic kidney disease; eGFR, estimated glomerular filtration rate.

Table 1 shows patients’ characteristics and correlations between the cerebral rSO_2_ and clinical parameters. Cerebral rSO_2_ showed significantly positive correlations with eGFR (Fig 2), Hb level, and serum sodium and albumin concentrations, in addition to the negative correlation with age, Ln-C-reactive protein, and Ln-urinary protein excretion.

**Table 1:** Patient characteristics and the correlation between cerebral rSO_2_ and clinical parameters in a simple linear regression analysis.

	mean ± SE	Simple linear regression	
vs. cerebral rSO_2_ values		r	*p* value
Number of patients (men/women)	40 (26/14)		
Cerebral rSO_2_ (%)	63.8 ± 1.5		
Age (years)	61.0 ± 2.7	-0.466	0.002
CKD stages G1/2/3a/3b/4/5	5/8/6/5/11/5		
Disease			
Chronic glomerulonephritis	24		
Nephrosclerosis	8		
Diabetes mellitus	2		
Others	6		
Body height (cm)	161 ± 1	0.123	0.449
Body weight (kg)	56.4 ± 2.3	0.218	0.175
Systolic blood pressure (mmHg)	136 ± 3	-0.212	0.187
Diastolic blood pressure (mmHg)	78 ± 2	0.211	0.190
Heart rate (/min)	75 ± 2	-0.270	0.091
Sat O_2_ (%)	97.3 ± 0.2	0.069	0.670
Laboratory findings			
Hb (g/dL)	12.0 ± 0.4	0.524	< 0.001
eGFR (mL/min/1.73m^2^)	46.6 ± 4.9	0.696	< 0.001
Na (mEq/L)	138 ± 1	0.479	0.002
K (mEq/L)	4.1 ± 0.1	-0.131	0.422
Cl (mEq/L)	103 ± 1	0.370	0.019
Ca (mg/dL)	9.2 ± 0.1	-0.274	0.087
P (mg/dL)	3.7 ± 0.1	-0.187	0.247
Total protein (g/dL)	6.7 ± 0.2	0.236	0.142
Serum albumin (g/dL)	3.6 ± 0.2	0.588	< 0.001
C-reactive protein (mg/dL), median (interquartile range)	0.2 (0.1–0.6)		
Ln-C-reactive protein	-1.7 ± 0.3	-0.410	0.008
Urinary protein excretion (g/g-Cr), median (interquartile range)	0.9 (0.4–2.5)		
Ln-urinary protein excretion	-0.5 ± 0.3	-0.448	0.004
Medication, *n* (%)			
Renin-angiotensin system blocker	16 (40.0)		
Calcium channel blocker	19 (47.5)		
Beta blocker	5 (12.5)		
Diuretics (loop and/or thiazide)	12 (30.0)		
Vitamin D analog	5 (12.5)		
Statin	8 (20.0)		
Antiplatelet agents	7 (17.5)		
Erythropoiesis-stimulating agent	5 (12.5)		

A multivariate linear regression analysis was performed using variables that showed a significant correlation with the cerebral rSO_2_ in a simple linear regression analysis (Table 2). The multivariate regression analysis showed that the cerebral rSO_2_ was independently associated with eGFR (standardized coefficient: 0.530), serum albumin concentration (standardized coefficient: 0.365), and serum sodium concentration (standardized coefficient: 0.224).

**Table 2:** Multivariable linear regression analysis: Independent factors of cerebral rSO_2_ in chronic kidney disease patients.

Variables	Coefficient	Standardized coefficient	*p*
eGFR	0.167	0.530	< 0.001
Serum albumin concentration	3.629	0.365	0.001
Serum sodium concentration	0.539	0.224	0.031
Ln-C-reactive protein	0.114	0.078	0.501
Ln-urinary protein excretion	-0.080	-0.057	0.637
Hb concentration	-0.054	-0.043	0.762
Age	-0.018	-0.014	0.954

eGFR, estimated glomerular filtration rate; Hb, hemoglobin.

**Fig 3:**
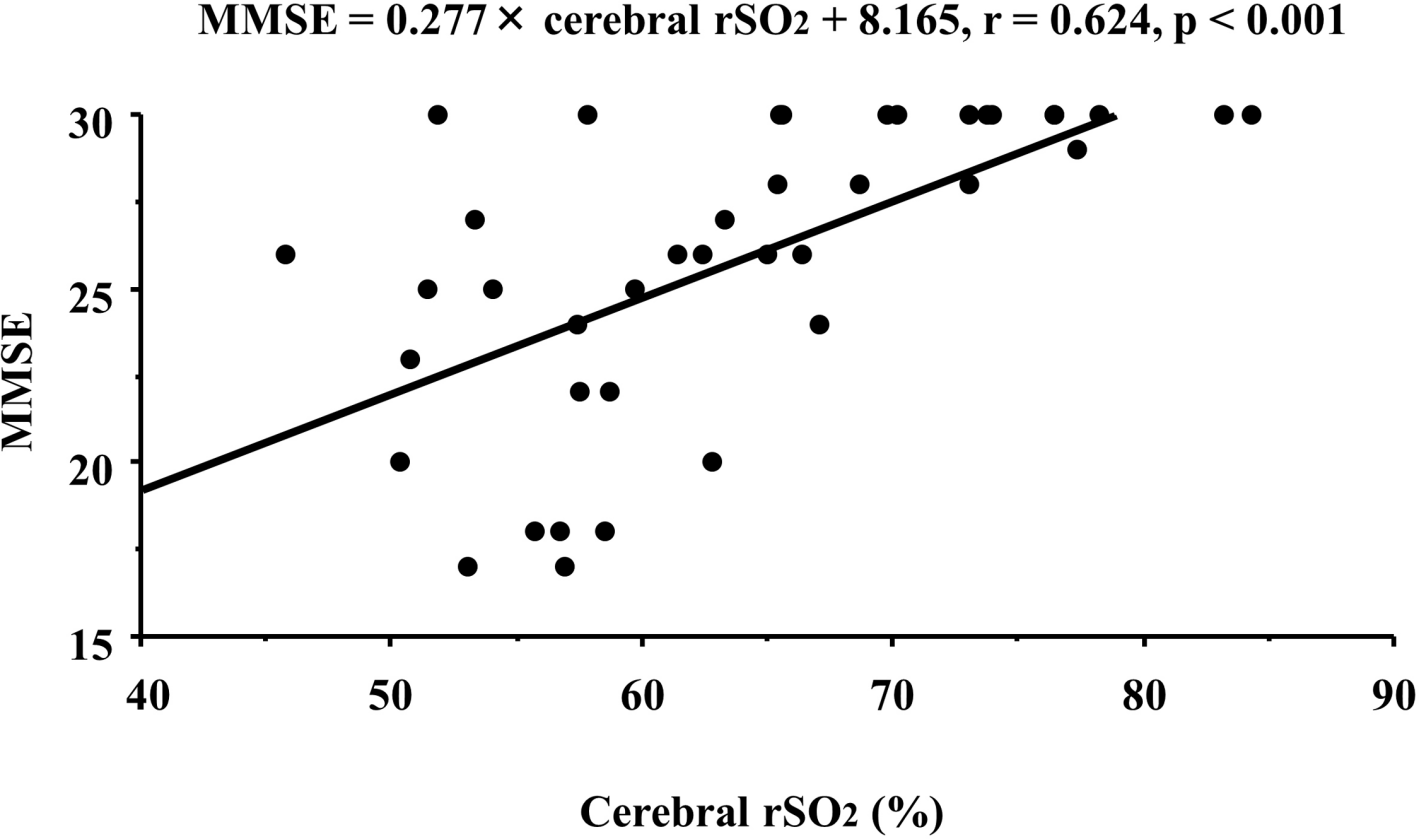
Correlation between MMSE and cerebral rSO_2_ in CKD patients. MMSE, Mini-Mental State Examination; rSO_2_, regional saturation of oxygen; CKD, chronic kidney disease.

Furthermore, the relationship between cerebral rSO_2_ and MMSE, as a marker of cognitive impairment, was evaluated in this study. MMSE scores showed a significantly positive correlation with cerebral rSO_2_ (r = 0.624, *p* < 0.001; Fig 3).

## Discussion

The present study first focused on the association between cerebral oxygenation and clinical parameters in CKD patients without dialysis and confirmed that cerebral rSO_2_ levels were affected by eGFR and serum albumin and sodium concentrations. Tissue rSO_2_ measurements using NIRS typically reflect the oxygen saturation in venous (70–80%), capillary (5%), and arterial blood (20–25%) [25]. Cerebral rSO_2_ values in healthy persons were reportedly nearly 70% and those in HD patients were significantly lower than those in healthy controls [12,13]. Furthermore, cerebral rSO_2_ values in HD patients were independently associated with pH, HD duration, serum albumin concentration, and DM [13].

Among the modifiable factors identified as being independently associated with cerebral rSO_2_, eGFR itself was the most potent factor affecting cerebral rSO_2_. In general, anatomical and hemodynamic features are similar in the brain and kidney [2]; therefore, the impairment of cerebral microcirculation represented by rSO_2_ decrease and eGFR values might be associated with common pathogenesis, including vascular injury, which leads to tissue ischemia in the brain and kidney. Concerning the vascular injury in CKD patients, the onset of CKD, which even occurred in early stages, stimulated aortic calcification in rat CKD models with mild or moderate renal injury [26], and aortic arch calcification was not only a consequence of CKD but also a predictor of renal function progression in CKD G3 to G5 patients [27]. Furthermore, recently in HD patients, cerebral rSO_2_ significantly decreased according to the progression of aortic arch calcification because of a decreased oxygen supply in the brain via cerebral macro- and/or microcirculation impairments [28]. Thus, even in the CKD patients enrolled in this study, cerebral rSO_2_ deterioration might have occurred due to cerebral vascular injury, including calcification associated with CKD progression itself, although we could not comment on the association between cerebral oxygenation and vascular injury with CKD patients because no examination was conducted to investigate vascular injury or calcification in this study.

Serum albumin concentration, the main determinant of colloid osmotic pressure in vessels, plays an important role in the regulation of fluid exchange across the microvascular wall. Therefore, albumin itself plays an essential role in maintaining microcirculation in systemic tissues via the body- fluid movement, mainly between the vessels and interstitium [29]. To date, serum albumin concentration has reportedly been associated with CKD progression [30], prognosis in CKD patients [31], and trajectory of cognitive function in community-dwelling adults [32]. Moreover, the assessment of the cognitive function subscale of the Kidney Disease Quality of Life showed the possibility to have an association between cognitive function and serum albumin concentration in CKD patients, including those undergoing HD [33]. In HD patients, serum albumin concentration has been reported to be independently associated with tissue oxygenation in the brain and lower-limb muscle [13,34]. Our result, that serum albumin concentration significantly influences cerebral rSO_2_ values in CKD patients without dialysis, is in concordance with that of previous report [13].

Furthermore, serum sodium concentration was a significant and positive factor affecting cerebral rSO_2_ values, even under a mean serum sodium concentration of 138 ± 1 mEq/L (127–144 mEq/L) in the present study. In the clinical setting, according to the CKD progression, loop- or thiazide- diuretics are frequently necessary to be used for the body- fluid management and are likely to cause hyponatremia, although these agents were administered in only 30% of CKD patients in the present study. Chronic hyponatremia has been associated with cognitive dysfunction and dementia because of the hyponatremia- inducing activation of renin- angiotensin- system, increases in inflammatory cytokines and oxidative stress, and decreases in ATP production [35,36]. In addition, in a model of female rats, chronic hyponatremia reportedly reduced cerebral blood flow by, in part, suppressing of basal nitric oxide- and prostacyclin- dependent tone in cerebral microcirculation [37,38]. Thus, the deterioration of cerebral rSO_2_ values might be associated with hyponatremia-associating cerebral microcirculation impairment. Therefore, at least, hyponatremia in CKD patients should be prevented from the perspective of cerebral oxygenation based on this study.

In contrast, Hb concentration did not show a significant association with cerebral rSO_2_ values in multivariable linear regression analysis, although a significant positive correlation was observed between the 2 in a simple linear regression analysis. In general, Hb itself plays an important role as an oxygen supplier to the systemic tissues and is expected to favorably influence the cerebral function by improving cerebral oxygenation. Indeed, in CKD patients, a lower Hb concentration was reportedly associated with a higher prevalence of cognitive impairment in a cross-sectional study [39]. Contrastingly, anemia was not independently associated with baseline cognitive function or decline in CKD patients [40]. Moreover, no relationship was found between Hb concentration and cerebral oxygenation, represented by cerebral rSO_2_ values, in HD patients with well-maintained Hb concentration [13]. Thus, to date, the relationship between Hb levels and brain function, including cognition and cerebral oxygenation, remains uncertain. Therefore, further studies are warranted to investigate the influence of Hb concentration on the brain function.

In addition to the evaluation of the association between cerebral rSO_2_ and clinical parameters, we investigated the correlation between cerebral rSO_2_ and MMSE scores and a significant and positive correlation was observed between the two parameters. Based on this result, cognitive function would worsen with the deterioration of cerebral oxygenation in CKD patients. In addition to not involving instruments to evaluate cognitive screening in CKD patients, cognitive function testing requires trained personnel and can be time consuming [2,33]; therefore, from the perspective of developing a simple method for evaluating cognitive function instead of formal cognitive testing, such as the MMSE, the measurements of cerebral rSO_2_ using NIRS technology might be useful and valuable in a clinical setting of CKD management. Furthermore, cognitive function was reportedly influenced by some medications in elderly or CKD patients [41,42]. Renin-angiotensin system blockers, including angiotensin-converting enzyme inhibitor and angiotensin II receptor blocker, have favorable effects in preventing cognitive decline via the improvement of the blood-brain barrier function, cerebral blood flow increase, inflammation reduction, and the reduction of amyloid-β peptide accumulation in the brain [41]. In addition, vitamin D favorably affects the cognitive function via the inhibition of oxidative stress, and increases antioxidant production, and prevents amyloid-β peptide accumulation [42]. These agents would be considered frequently essential in the strategy of CKD treatment. In this study, renin-angiotensin system blockers and vitamin D analog were administered to 40% and 12.5% of the patients, respectively. However, we cannot comment on the association between cognitive function and the administration of renin-angiotensin system blockers and/or vitamin D analog because of the small sample size in this study.

This study has several limitations. First, its sample size was relatively small. Second, we examined the association of cerebral rSO_2_ with only clinical parameters, including eGFR, and we could not evaluate the differences of cerebral rSO_2_ values in each disease, such as chronic glomerulonephritis, nephrosclerosis, and DM, which cause the deterioration of renal function. Furthermore, we did not routinely examine the cerebral vascular status of CKD patients without neurological disorders. Thus, we could not evaluate the injury or calcification of cerebral vessels and comment on the association between cerebral oxygenation and vascular status in this study. Finally, only MMSE was performed as a cognitive assessment in this study. Cognition is well-known to be classified into several domains including visuo-spatial perception, auditory memory, visual memory, attention span, motor function, and mathematical reasoning. Therefore, further studies are warranted to comprehensively elucidate the association between cerebral rSO_2_ and various clinical parameters with other cognitive tests, including the evaluation of differentiated specific aspects of cognition.

In conclusion, cerebral rSO_2_ was affected by eGFR and serum albumin and sodium concentrations in CKD patients. Furthermore, cerebral rSO_2_ monitoring, which reflected MMSE scores, might be a useful method for assessing cognitive function in CKD patients.
